# New adaptive lasso approaches for variable selection in automated pharmacovigilance signal detection

**DOI:** 10.1186/s12874-021-01450-3

**Published:** 2021-12-01

**Authors:** Émeline Courtois, Pascale Tubert-Bitter, Ismaïl Ahmed

**Affiliations:** High-Dimensional Biostatistics for Drug Safety and Genomics, CESP, Université Paris-Saclay, UVSQ, Université Paris-Sud, Inserm, Villejuif, France

**Keywords:** Adaptive logistic lasso, BIC, Variable selection, Drug safety signal, Spontaneous reporting

## Abstract

**Background:**

Adverse effects of drugs are often identified after market introduction. Post-marketing pharmacovigilance aims to detect them as early as possible and relies on spontaneous reporting systems collecting suspicious cases. Signal detection tools have been developed to mine these large databases and counts of reports are analysed with disproportionality methods. To address disproportionality method biases, recent methods apply to individual observations taking into account all exposures for the same patient. In particular, the logistic lasso provides an efficient variable selection framework, yet the choice of the regularization parameter is a challenging issue and the lasso variable selection may give inconsistent results.

**Methods:**

We propose a new signal detection methodology based on the adaptive lasso. We derived two new adaptive weights from (i) a lasso regression using the Bayesian Information Criterion (BIC), and (ii) the class-imbalanced subsampling lasso (CISL), an extension of stability selection. The BIC is used in the adaptive lasso stage for variable selection. We performed an extensive simulation study and an application to real data, where we compared our methods to the existing adaptive lasso, and recent detection approaches based on lasso regression or propensity scores in high dimension. For both studies, we evaluate the methods in terms of false discoveries and sensitivity.

**Results:**

In the simulations and the application, both proposed adaptive weights show equivalent or better performances than the other competitors, with an advantage for the CISL-based adaptive weights. CISL and lasso regression using BIC are solid alternatives.

**Conclusion:**

Our proposed adaptive lasso is an appealing methodology for signal detection in pharmacovigilance. Although we cannot rely on test theory, our approaches show a low and stable False Discovery Rate in all simulation settings. All methods evaluated in this work are implemented in the adapt4pv R package.

**Supplementary Information:**

The online version contains supplementary material available at (10.1186/s12874-021-01450-3).

## Background

Because the conditions of exposure of an active drug in real life are very different from those of clinical trials, the adverse effects of drugs are often identified once they are introduced on the market. This may be due to a complex interaction with subcategories of population, or to a long latency period after exposure. Post-marketing pharmacovigilance aims to detect as early as possible these adverse effects that have not been identified during the safety assessment stages of drug development. Pharmacovigilance systems rely on large databases of individual case safety reports of adverse events (AEs) suspected to be drug-induced. Many countries currently have a spontaneous reporting system as well as supranational entities such as the European Medicines Agency or the Uppsala Monitoring Centre in charge of pharmacovigilance for the World Health Organization. In France, the national pharmacovigilance database is maintained by the National Agency for the Safety of Drugs and Health Products (*Agence Nationale de Sécurité du Médicament et des Produits de Santé*, ANSM). It contained around 450 000 reports at the end of December 2017. Currently, about 36 000 reports are reported annually.

Several automated signal detection tools have been developed to mine these large amounts of data in order to highlight suspicious AE-drug combinations. To draw definite conclusions, these signals need further expert investigations or additional studies. This is why it is important to generate a reasonable number of signals with as few false associations as possible for further analysis. Thus, performances of these signal detection approaches are evaluated according to their ability to identify toxicities truly associated with drugs as well as their ability to not generate a signal when there are no relationship between a drug and an adverse event. Classical signal detection methods are based on disproportionality analyses of counts aggregating patients’ reports for each drug-AE pair [1–4]. These methods have been extended to account for multiple comparison testing in order to provide alternative signal ranking and detection thresholds based on false discovery rate (FDR) estimates [5–7]. Other methods for aggregated counts relying on likelihood ratio tests have also been proposed [8, 9].

Disproportionality methods are subject to the masking effect bias and do not account for co-prescription [10–13]. In recent years, multiple logistic regression-based signal detection methods which rely on lasso penalization [14, 15] have been proposed to address these limitations. Unlike disproportionality methods, they are directly applied to individual spontaneous reports rather than to aggregated counts. For an observation, the outcome is the presence or absence of a given AE, and the covariates are all drug presence indicators. The objective of pharmacovigilance therefore pertains to the variable selection framework by aiming to identify the drugs potentially associated with AE among the multitude of candidate covariates. The drug exposure matrix is thus large, binary and extremely sparse, and there is also a large imbalance between the presence and absence of a given AE. More recently, signal detection methods based on propensity scores (PS) in high dimension have also been proposed as an alternative to address disproportionality method biases [16–18].

Lasso penalization is a computationally efficient way to perform regression in high dimension [19]. The parsimony induced by the *L*_1_ norm is also an appealing feature of this algorithm. Nevertheless, while cross-validation is classically used for the purpose of prediction, it is less straightforward to choose the best regularization parameter controlling the sparsity of the model in the variable selection framework. Furthermore, it has been shown that there is no proper regularization parameter that allows the lasso to enjoy the oracle properties defined by Fan and Li [20]. This means for instance that the lasso variable selection may be inconsistent. Subsampling strategies such as stability selection [21] have been proposed to lessen the importance of the choice of regularization parameter, and the class-imbalanced subsampling lasso (CISL) [15] was specifically designed to account for the large imbalance in spontaneous reporting data.

The adaptive lasso is an alternative approach to improve the variable selection properties of the lasso [22]. It consists in using adaptive weights (AWs) for penalizing covariates differently in the *L*_1_ penalty. Originally, AWs were derived from coefficients estimated by maximum likelihood. A high-dimensional version of the adaptive lasso has been proposed by Bühlmann and Van De Geer [23] in the linear case, in which the AWs are derived from the coefficients obtained by a first lasso regression. Huang et al. [24] proposed the same approach in the logistic case. They also showed in another work that in the linear case, AWs derived from univariate regression coefficients result in good recovery properties under certain conditions [25]. To our knowledge, the adaptive lasso has never been used for signal detection in pharmacovigilance.

In this work, we present a new automated signal detection strategy based on the adaptive lasso which aims at improving the guidance of the variable selection operated by the lasso through adaptive penalty weights specific to each covariate. This new strategy also involves the use of the Bayesian Information Criterion (BIC). We propose two new AWs derived from (i) a lasso logistic regression for which the regularization parameter is chosen using the BIC, and (ii) CISL. These AWs are then incorporated into a lasso logistic regression using the BIC to choose the regularization parameter. We compare both versions of our approach to (i) more classical implementations of the adaptive lasso in high dimension [23–25], (ii) lasso regressions considering cross-validation, BIC or permutations [26, 27] for choosing the regularization parameter, (iii) CISL and (iv) the propensity score in high dimension-based approaches that were recently proposed. We conducted an extensive simulation study exploiting real drug exposure data from the French pharmacovigilance database in order to preserve the sparsity of the covariates. We also present an empirical study on the French national database using a large and recently published reference set pertaining to drug-induced liver injuries (DILI) [28, 29]. Performances of all the presented methods are evaluated in terms of false discoveries and sensitivity.

## Methods

We first present the lasso-based detection approaches. Then we detail the detection approaches based on the propensity score in high dimension. In a third step, we detail implementations of adaptive lasso proposed in the literature and we present our proposals based on the adaptive lasso.

### The logistic lasso

Let *N* denote the number of spontaneous reports (i.e. the number of observations) and *P* the total number of drug covariates. Let **X** denote the *N*×*P* binary matrix of drug exposures and let *x*_*i*_ be a 1×*P* vector of covariates for the *i*th observation. Let **y** be the *N*-vector of binary responses that indicates the presence or absence of the AE of interest. For *i*∈{1,...,*N*}, the corresponding multiple logistic model is

1 $$  \text{logit}(\text{Pr} (y_{i} = 1 | \mathbf{x_{i}})) = \beta_{0} + \sum_{p=1}^{P} \beta_{p} ~ x_{{ip}},  $$

where *β*_0_ is the intercept and ***β*** is a *P*-vector of regression coefficients associated with drug covariates. Although we are not in the *P*>>*N* context, *P* is typically very large, which can cause some numerical problems with classical regression. The penalized logistic lasso consists in estimating:

$$\begin{array}{*{20}l} \left(\widehat{\beta_{0}}_{\lambda}, \widehat{\boldsymbol{\beta}}_{\lambda}\right) = \text{argmax}_{\left(\beta_{0}, \boldsymbol{\beta}\right)} \left\{ l\left(\left(\beta_{0}, \boldsymbol{\beta}\right), \mathbf{y}, \mathbf{X}\right) - \text{pen}(\lambda) \right\}, \end{array} $$

where *l* is the log-likelihood of model (1), *λ* is the regularization parameter and pen(*λ*) is defined as

2 $$  \text{pen}(\lambda) = \lambda | \boldsymbol{\beta} |_{1} = \lambda \sum_{p=1}^{P} | \beta_{p} |.  $$

Thanks to the *L*_1_ penalty in (2), some coefficients of $\widehat {\boldsymbol {\beta }}_{\lambda }$ are shrunk to exactly zero, so the covariates associated with these coefficients are not retained in the model. By controlling the amount of penalization, the *λ* parameter in the lasso regression is closely related to the number of non-zero estimated coefficients. Since the aim in pharmacovigilance is to detect deleterious associations with the outcome, we are only interested in covariates with a positive associated penalized coefficient in $\widehat {\boldsymbol {\beta }}_{\lambda }$.

#### Penalization parameter selection

We considered three strategies for selecting the penalization parameter: cross-validation, BIC and permutations. One round of cross-validation involves partitioning the dataset into *n*_*f*_ subsets, called folds: *n*_*f*_−1 are used as a training set, i.e. the model is estimated on this set, and the remaining fold is used as a validation set where a prediction performance metric (e.g. area under curve or deviance) is calculated. This procedure is repeated so that each fold is used only once as a validation set. An average value of the performance metric and a standard deviation are then calculated over the *n*_*f*_ obtained values. In the lasso regression context, cross-validation is performed for each tested *λ* value. The selected *λ* according to cross-validation is the one with the best result in terms of the prediction performance metric selected. In this work, we used the deviance, and we set *n*_*f*_ to 5.

An alternative strategy for selecting the penalization parameter is to rely on model selection criteria such as the BIC [15, 27]. For each tested *λ*, we implemented the BIC as follows:

3 $$  \text{BIC}_{\lambda} = - 2 l_{\lambda} + \text{df}(\lambda) ~ \text{ln} (N),  $$

where *l*_*λ*_ is the log-likelihood of the classical multiple logistic regression model, which includes the set of covariates with a non-zero coefficient in $\widehat {\boldsymbol {\beta }}_{\lambda }$, and $\text {df}(\lambda) = | \widehat {\boldsymbol {\beta }}_{\lambda } \neq 0 |$. If different *λ*s lead to the same subset of retained covariates, then the non-penalized models resulting from these *λ*s are the same, as is the BIC. Consequently, this approach selects the subset of covariates that leads to the classical model which minimizes the BIC defined in (3), rather than selecting a particular *λ*.

An approach based on permutations for selecting the penalization parameter in lasso regression was proposed by Sabourin et al. [27] based on the suggestion of Ayers and Cordell [26]. Denoting *π* as any permutation of {1,...,*N*}, let $\phantom {\dot {i}\!}\mathbf {y}_{\pi _{l}} = (y_{\pi (1)},..., y_{\pi (N)})$ be a permuted version of the outcome **y** with 1≤*l*≤*K*. A lasso regression is performed for each of these permutations by regressing $\phantom {\dot {i}\!}\mathbf {y}_{\pi _{l}}$ on the original data set **X**. One then obtains $\phantom {\dot {i}\!}\lambda _{{max}}(\mathbf {y}_{\pi _{l}})$, i.e. the smallest value of the penalty parameter, such that no covariate is selected in the lasso regression on $\phantom {\dot {i}\!}\mathbf {y}_{\pi _{l}}$. As in Sabourin et al. [27], we used the median value of $\phantom {\dot {i}\!}\left (\lambda _{{max}}(\mathbf {y}_{\pi _{1}}),..., \lambda _{{max}}(\mathbf {y}_{\pi _{K}})\right)$ in a lasso regression performed with the original outcome **y**. In this work, we set *K*=20.

In the following, we refer to the approach involving cross-validation, BIC or permutation to choose the penalty parameter as lasso-cv, lasso-bic and lasso-perm, respectively.

#### Class-imbalanced subsampling lasso

To circumvent the penalization parameter selection issue in lasso regression, Meinshausen and Bühlmann proposed the stability selection algorithm [21]. Briefly, it consists of perturbing the data by subsampling many times, implementing lasso regression on these subsamples randomly drawn without replacement, and choosing covariates that occur in a large fraction of the resulting selected sets induced by the lasso path of regularization. Ahmed et al. proposed a variation of this method to account for the large imbalance of the outcome that occurs in pharmacovigilance databases: the CISL algorithm [15]. In CISL, subsamples are drawn following a nonequiprobable sampling scheme with replacement in order to allow a better representation of individuals who experienced the outcome of interest. Lasso regressions are performed in each of these samples and the following quantity is computed:

4 $$  \widehat{\pi}^{b}_{p}= \frac{1}{E} \sum_{\eta = 1}^{E} \mathbbm{1} \left[ \widehat{\beta}_{p}^{\eta, b} > 0 \right],  $$

where *E* is the maximum number of covariates selected by all the lasso regressions, *η*∈{1,..,*E*} is the number of covariates selected and $\widehat {\beta }^{\eta, b}_{p}$ is the regression coefficient estimated by the logistic lasso for drug *p*, on sample *b*∈{1,..,*B*} for a model including *η* covariates. Thus, for each drug, an empirical distribution of $\widehat {\pi }^{b}_{p}$ is obtained over all *B* samples. The drug covariate is then selected if a given quantile of the distribution of $\widehat {\pi }^{b}_{p}$ is non-zero. In this work, we considered the covariate sets established with the 10% quantiles of these distributions following Ahmed et. al.’s recommendation.

### Propensity score approaches

The propensity score (PS) is defined as the probability of being exposed to a drug of interest given the observed covariates [30]. It is a balancing score, which means that conditionally on the PS, treatment exposure and the observed covariates are independent, so it is possible to deal with measured confounding. Recently, this methodology was extended to exploit large healthcare databases. In this framework, covariate selection algorithms are used to automatically select potential confounders for inclusion in the PS estimation model of a given drug exposure [31].

In Courtois et al. [17], several PS-based approaches were proposed in the context of signal detection from spontaneous reporting data. These approaches consisted in estimating a PS for each drug reported in the database. The PSs were built by selecting among all the other drugs those to be included in the PS estimation model. Because of the large number of candidate covariates to be included in these models, covariate selection algorithms were used and compared. Here we used the lasso-bic approach presented earlier to select the set of covariates to be included in the PS logistic regression model. This procedure was repeated for all drugs in the database. Following Courtois et al, we accounted for these PSs in the final regression model through adjustment and weighting with two different weightings for the latter: Inverse Probability of Treatment Weighting (IPTW) [32] and Matching Weights (MW) [33]. We also investigated the weights truncation approach with IPTW. This consists in assigning to individuals whose corresponding weight is below the *r*th percentile or above the (1−*r*)th percentile of weights, the value of the *r*th or (1−*r*)th percentile, respectively [34]. Here we chose to set *r*=2.5*%*.

For a given PS-based approach, each drug was evaluated using one-sided hypothesis testing. To account for multiple testing, we used the procedure proposed by Benjamini and Yekutelli [35] to control the FDR under arbitrary dependence assumptions. We set the FDR level at 5%. In the following, we refer to the adjustment on the PS, the weighting on the PS with weights IPTW, IPTW with truncation, and MW as ps-adjust, ps-iptw, ps-iptwT and ps-mw, respectively.

### Adaptive lasso and extensions for signal detection

#### The adaptive lasso

As defined by Fan and Li [20], an optimal procedure in statistical learning should have the following oracle properties: (i) identifies the right subset of true predictors, and (ii) produces unbiased estimates. In their work, they showed that the lasso procedure does not enjoy these oracle properties. Indeed, there are some scenarios in which the lasso variable selection could be inconsistent. Furthermore, with an equal penalty for all covariates, the lasso tends to overpenalize the relevant ones and to produce biased estimates for true large coefficients. To overcome this drawback, Zou [22] proposed the adaptive lasso in which AWs are used to penalize covariates differently in the *L*_1_ penalty:

$$\begin{array}{*{20}l} \text{pen}(\lambda) = \lambda \sum_{p=1}^{P} w_{p} | \beta_{p} |. \end{array} $$

The penalty applied to the covariate *p* is defined by *λ*_*p*_=*λ*×*w*_*p*_. The higher the value of the weight *w*_*p*_, the more the variable *p* is penalized and the less likely the variable is to be included in the model. By assigning a higher penalty to small coefficients and a lower penalty to large ones, the adaptive lasso makes it possible to consistently select the right model and produce unbiased estimates. Thus, Zou showed in his work that under certain conditions for the AWs, the adaptive lasso enjoys the oracle properties.

To build the AWs, Zou used an initial consistent estimator of ***β***^∗^, the *P*-vector of regression coefficients. To this end, he considered $\widehat {\boldsymbol {\beta }}^{mle}_{p}$ the maximum likelihood estimate for covariate *p* and defined the associated penalty weight $w_{p} = \frac {1}{|\widehat {\beta }^{mle}_{p}|^{\gamma }}$, with *γ*>0. However, in the high-dimensional context, it is non-trivial to find a consistent estimate for constructing the AWs since computing the maximum likelihood is not feasible.

In the linear case, Bühlmann and Van De Geer [23] proposed to use the penalized regression coefficients estimated by a lasso regression to determine these AWs considering *γ*=1. In both the lasso and the adaptive lasso, the penalization parameter *λ* was selected through cross-validation. This two-stage procedure, which involves an initial lasso step with cross-validation, was proposed in the logistic case under the name of iterated lasso [24]. By denoting $\widehat {\boldsymbol {\beta }}^{lcv}$ the *P*-vector of lasso regression coefficients determined with lasso-cv, the AWs associated with the drug covariate *p* in the adaptive lasso stage are defined as:

$$\begin{array}{*{20}l} w^{lcv}_{p} = \left\{ \begin{array}{ccc} \frac{1}{|\widehat{\beta}_{p}^{lcv}|} & \text{if} & \widehat{\beta}_{p}^{lcv} \neq 0 \\ \infty & \text{if} & \widehat{\beta}_{p}^{lcv} = 0 \end{array}\right. \end{array} $$

Thus, a covariate that has not been selected with lasso-cv in the first stage is automatically excluded in the adaptive lasso stage. In the following, we refer to this approach as adapt-cv.

In the linear case, Huang et al. [25] showed that under certain conditions, using univariate regression coefficients to determine the AWs presents nice properties. By denoting $\widehat {\beta }^{univ}_{p}$ the univariate coefficient associated to drug covariate *p*, we defined the following AWs associated with the drug covariate *p* in the adaptive lasso stage:

$$\begin{array}{*{20}l} w^{univ}_{p} = \frac{1}{|\widehat{\beta}_{p}^{univ}|}. \end{array} $$

As in the work of Huang et al., we chose the penalisation parameter according to cross-validation in the adaptive lasso stage. We refer to this approach as adapt-univ.

Following Ballout et al. [36], the optimal *λ* for cross-validation-based adaptive lasso is obtained by deriving adaptive weights for each training set (i) directly for adapt-univ or (ii) using an embedded cross-validation for adapt-cv. This optimal *λ* is then used on the full data to obtain the final adaptive lasso estimates.

#### Extending adaptive lasso for pharmacovigilance

Although adaptive lasso is an appealing variable selection procedure, to our knowledge it has never been used for signal detection. Since the aim in pharmacovigilance is to select the right subset of drugs associated with an AE, we sought to develop a signal detection approach by enhancing the performance of this method in terms of variable selection. To this end, we first use the BIC as defined above to identify the final subset of covariates in the adaptive lasso stage instead of cross-validation. We also propose two new AWs that aim to under-penalise variables that have been considered relevant by lasso-based variable selection methods, and to increase the penalty applied to, or even exclude, variables considered as less relevant.

The first one consists in using the BIC in the first stage. By denoting $\widehat {\boldsymbol {\beta }}^{lbic}$ the *P*-vector of unpenalized regression coefficients estimated in the first stage with lasso-bic, we define the following AWs associated with the drug covariate *p* in the adaptive lasso stage by:

$$\begin{array}{*{20}l} w^{lb}_{p} = \left\{ \begin{array}{ccc} \frac{1}{|\widehat{\beta}_{p}^{lbic}|} & \text{if} & \widehat{\beta}_{p}^{lbic} \neq 0 \\ \infty & \text{if} & \widehat{\beta}_{p}^{lbic} = 0 \end{array}\right. \end{array} $$

A covariate that has not been selected by the lasso-bic in the first stage is automatically excluded in the adaptive lasso stage.

The second proposed AWs are derived from the CISL approach. We first compute CISL by considering a non-zero constraint in calculating of the quantity (4) instead of the original positive constraint:

$$\begin{array}{*{20}l} \widehat{\tau}^{b}_{p}= \frac{1}{E} \sum_{\eta = 1}^{E} \mathbbm{1} [ \widehat{\beta}_{p}^{\eta, b} \neq 0 ]. \end{array} $$

This quantity measures the proportion to which a variable has been selected in *E* first models provided by the lasso regularization path. We define AW for covariate *p* according to the *B*-vector $\widehat {\boldsymbol {\tau }}_{p}$ as:

$$\begin{array}{*{20}l} w^{cisl}_{p} = \left\{ \begin{array}{ccc} \frac{1}{B} & \text{if} & \forall b \in \{1,..., B\} ~ \widehat{\tau}^{b}_{p} >0 \\ \\ \infty & \text{if} & \forall b \in \{1,..., B\} ~ \widehat{\tau}^{b}_{p} = 0 \\ \\ 1- \frac{1}{B} \sum_{b=1}^{B} \mathbbm{1} [ \widehat{\tau}^{b}_{p} >0 ] & & \text{otherwise.} \end{array}\right. \end{array} $$

Thus, the more $\widehat {\boldsymbol {\tau }}_{p}$ is non-null over the *B* subsamples, the smaller is its associated AW.

In the following, we refer to these approaches as adapt-bic and adapt-cisl.

## Simulation study

We performed a simulation study to assess the performances of the proposed adaptive lasso strategies and to compare all the methods described above. We investigated a large number of scenarios in terms of event prevalence, number of true signals, exposure frequency and strength of association. We compared the ability of each method to detect true signals and not detect false signals through sensitivity and FDR.

### Comparison set-up

We simulated the occurrence of a given AE according to a logistic regression model *y*_*i*_∼Bernoulli(*α*_*i*_) with $\alpha _{i} = \frac {1}{1 + \text {exp}\left (- \beta _{0} - \sum _{p=1}^{P} \beta _{p} x_{{ip}} \right) }$. As for the drug exposure matrix, we used the French pharmacovigilance database for the period 2000-2017 which contains 452 914 individual reports and 2 378 different drugs (see “Real-world data analysis” section for a description of the data). For each replication of each scenario, we first randomly selected 100 000 individual reports out of the 452 914 individual reports. For each of these datasets, we then randomly selected a subset of 500 drugs among those reported more than 10 times. Thus, for each simulation scenario, *N* and *P* were set at 100 000 and 500 respectively. We investigated 27 scenarios that differed according to:

- the value of the intercept *β*_0_, the latter being used to simulate outcomes of varying scarcity *β*_0_∈{−2,−4,−6};
- the number of drugs associated with the outcome among the 500 drug covariates: *n*_*TP*_∈{0,5,20};
- the value of the regression coefficients for the *n*_*TP*_ true predictors: *β*_*TP*_∈{1,2};
- the reporting frequency of the true predictors (if any): frequent (at least 100 reports over 100 000) or rare (between 20 and 100 reports over 100 000).

Note that for each scenario, the *n*_*TP*_ true predictors were chosen randomly for each of the 500 replications.

In order to measure the relevance of using the BIC with the adaptive lasso, we included a method based on the same AWs as adapt-univ in the comparison. However, instead of cross-validation, we used the BIC to perform variable selection in the adaptive lasso stage. In the following we refer to this approach as adapt-univ-bic. For the sake of clarity, Table 1 summarizes all the implemented signal detection approaches based on the adaptive lasso. For each approach, it details how the AWs are obtained and what variable selection method is used in the adaptive lasso stage.

**Table 1:** Characteristics of signal detection approaches based on adaptive lasso

Method	Construction of adaptive weights	Criterion for variable selection in adaptive lasso stage
adapt-cv	lasso-cv	cross-validation
adapt-univ	univariate coefficients	cross-validation
adapt-univ-bic	univariate coefficients	BIC
adapt-bic	lasso-bic	BIC
adapt-cisl	CISL	BIC

We declared as signals all drugs positively associated with the outcome for all the lasso and adaptive lasso-based approaches. For PS-based approaches, we applied a supplementary filter by considering only drugs which had more than three reports in common with the outcome. Drugs discarded by the filter had their associated p-value set to one [37]. For the sake of completeness, we also included a disproportionality method in the comparison: the Reporting Fisher’s Exact Test (RFET) [5]. Compared to the more classical Reporting Odds Ratio (ROR) and Proportional Reporting Ratio (PRR), the RFET does not rely on asymptotic assumptions which are often not met given the low number of observed counts. As for the PS-based approaches, RFET was implemented on drugs with more than three reports in common with the outcome, and one-sided p-values were considered. We applied the multiple testing correction procedure to RFET presented in the “Propensity score approaches” section.

In total, we compared 14 signal detection approaches: one disproportionality method, four lasso-based approaches, five adaptive lasso-based approaches and four PS-based approaches. All these approaches (except RFET) are implemented in the R package adapt4pv available on the CRAN. All the analyses were performed with R version 3.6.0. All the logistic regressions were computed with the speedglm R package v0.3-2 designed to handle sparse matrices efficiently. All lasso regressions were implemented using the glmnet R package v3.0-2.

### Results

Table 2 shows the average number of drug covariates and the average number of true predictors kept after discarding covariates with fewer than three reports in common with the simulated outcome per scenario. Table 2 also shows the average number of cases per scenario. As the number of cases decreased, the number of covariates retained after filtering decreased, which also included true predictors. When the true predictors were rarely reported and the outcome was particularly rare, there were no true predictors retained after filtering (scenarios 24, 25, 26).

**Table 2:** Average number of drug covariates/ true predictors retained after filtering and average number of cases according to scenario settings

Scenario	*β*_0_	*n*_*TP*_	*β*_*TP*_	Average number of covariates retained after filtering	Average number of true predictors retained after filtering	Average number of cases
**No true predictors**						
1	-2	0	0	389	NA	11 923
2	-4	0	0	165	NA	1 798
3	-6	0	0	30	NA	247
**True predictors reported more than 100 times**						
4	-2	5	1	394	5	12 327
5	-2	5	2	400	5	12 937
6	-2	20	1	407	20	13 589
7	-2	20	2	424	20	15 956
8	-4	5	1	172	5	1 879
9	-4	5	2	184	5	2 083
10	-4	20	1	193	20	2 155
11	-4	20	2	237	20	3 121
12	-6	5	1	34	2	259
13	-6	5	2	44	4	294
14	-6	20	1	49	11	300
15	-6	20	2	95	18	507
**True predictors reported between 20 to 100 times**						
16	-2	5	1	391	5	11 958
17	-2	5	2	392	5	12 013
18	-2	20	1	396	20	12 064
19	-2	20	2	400	20	12 284
20	-4	5	1	167	2	1 805
21	-4	5	2	171	4	1 822
22	-4	20	1	175	8	1 827
23	-4	20	2	191	17	1 897
24	-6	5	1	30	0	248
25	-6	5	2	31	0	251
26	-6	20	1	31	0	251
27	-6	20	2	35	2	263

**Fig. 1:**
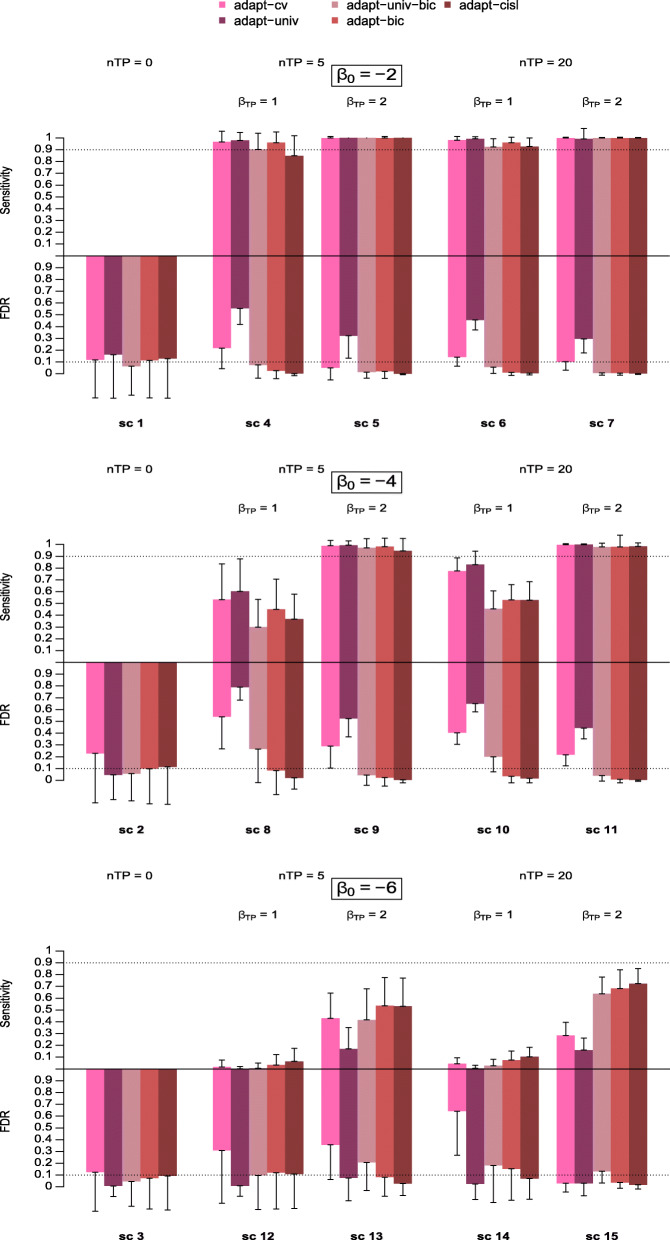
Sensitivity and False Discovery Rate of signal detection approaches based on adaptive lasso across scenarios 1 to 15. The upper and lower parts of the colour bars represent the average sensitivity and FDR of each approach over the 500 simulation replications respectively. The vertical solid lines extending the bars represent the standard deviation of the corresponding metrics

We first compared the performances of our proposed approaches versus the other adaptive lasso-based approaches. Figure 1 shows the average FDR and sensitivity (across the 500 replications) of the approaches listed in Table 1 for scenarios 1 to 15, i.e. scenarios in which there are no true predictors (scenario 1-3) and scenarios with true predictors frequently reported (scenario 4-15). Standard deviations of these metrics over the 500 simulation replications are also shown for each approach and each scenario. All adaptive lasso-based detection approaches showed low FDR for scenarios 1-3, with slightly worse performance for adapt-cv. In scenarios where *β*_0_=−2 and *β*_0_=−4 (scenarios 4 to 7 and 8 to 11) adapt-cv and adapt-univ showed a high sensitivity at the cost of a high FDR, especially for adapt-univ. This is particularly the case in scenarios 8 and 10. For scenarios 12 to 15, where the outcome is rare (*β*_0_=−6), these two approaches showed a lower sensitivity. In these scenarios, adapt-univ showed a low FDR while adapt-cv had an unstable behaviour in terms of FDR, with a high average FDR in scenarios 12 to 14, and very low in scenario 15. By comparing adapt-univ and adapt-univ-bic, we find that using the BIC to perform variable selection in the adaptive lasso step reduced the FDR for *β*_0_=−2 and *β*_0_=−4. For *β*_0_=−6, this is not the case, adapt-univ showing particularly low FDR and low sensitivity in this setting. By comparing approaches which rely on BIC in the adaptive lasso stage, namely adapt-univ-bic, adapt-bic and adapt-cisl, we see that approaches based on our proposed AWs performed better overall, since they showed both a lower FDR and a higher sensitivity than adapt-univ-bic. These differences in performance were particularly noticeable in scenarios 4, 8, 10, and 13 to 15. In scenarios 12 to 15, adapt-bic and adapt-cisl showed a better sensitivity than approaches based on cross-validation with a lower FDR. Nonetheless, adapt-univ-bic had a slightly lower FDR than adapt-bic and adapt-cisl when there were no true predictors (scenarios 1-3), with an FDR around 0.10 for adapt-cisl and adapt-bic, and around 0.05 for adap-univ-bic.

Among our proposals, adapt-cisl generally performed better than adapt-bic with a lower FDR and a slightly higher sensitivity, in particular in scenarios 12 to 15 when the outcome was rare. In these scenarios, the FDR of adapt-cisl ranged from 0.01 to 0.10 and its sensitivity ranged from 0.06 to 0.72, while the FDR of adapt-bic ranged from 0.03 to 0.12 and its sensitivity ranged from 0.03 to 0.68.

**Fig. 2:**
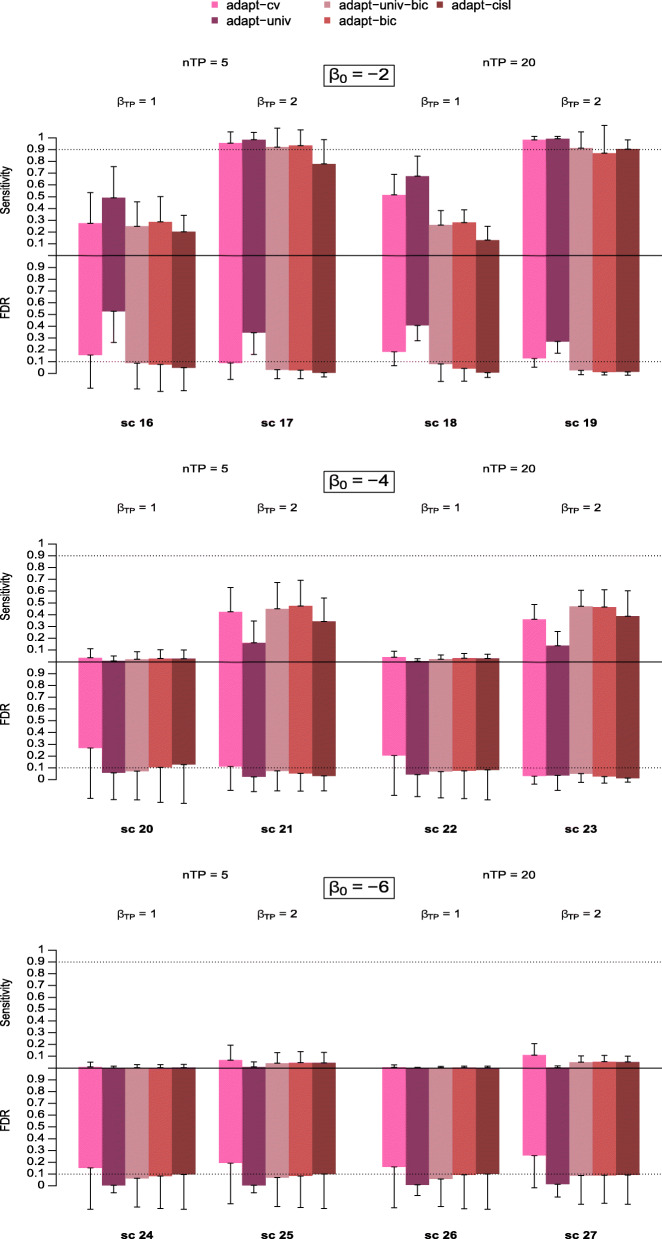
Sensitivity and False Discovery Rate of signal detection approaches based on adaptive lasso across scenarios 16 to 27. The upper and lower parts of the colour bars represent the average sensitivity and FDR of each approach over the 500 simulation replications respectively. The vertical solid lines extending the bars represent the standard deviation of the corresponding metrics

Simulation results for scenarios 16 to 27, i.e. for true predictors reported between 20 and 100 times, are shown in Fig. 2 for all these approaches. Unsurprisingly, all the approaches showed a lower sensitivity in these scenarios compared to scenarios 4 to 15.

Overall, although the differences in performance are less clear-cut than in Fig. 1, the behaviour of the different approaches is quite similar. For scenarios where the outcome is frequent (scenarios 16-19) adapt-univ showed a high sensitivity and a rather high FDR. At the opposite, as the outcome became rarer this approach became very conservative with a low FDR and a low sensitivity. Adapt-cv tended to show a higher FDR (e.g scenarios 20 and 22) and a higher sensitivity (e.g. scenarios 18, 25, 27) than the three BIC-based approaches. Adapt-univ-bic showed a lower FDR when the true predictors were less reported. Overall our proposals adapt-cisl and adapt-univ performed the best, with an FDR that remained low and a good sensitivity, as in scenarios 1 to 15, and a stable performance behaviour across all simulation scenarios.

For all methods, the variability in the FDR estimation over the 500 replications was greater in scenarios where *n*_*TP*_=0 (i.e. scenarios 1, 2 and 3). It decreased with the intercept value for all the approaches. Overall, our proposals showed more stable results in terms of FDR compared to adapt-cv, adapt-univ and adapt-univ-bic with lower or equal standard deviations. In terms of sensitivity, all approaches presented results of comparable standard deviations in the majority of scenarios, except in some scenarios where our approaches showed greater standard deviations. This is particularly the case in scenario 19 for adapt-bic.

In supplementary materials, Table A shows the average number of signals generated by adapt-cv, adapt-univ, adapt-univ-bic, adapt-bic, adapt-cisl across all the scenario settings. For all these approaches, as the outcome and true predictors became rarer, the number of signals generated decreased. When the outcome is frequent, adapt-cv and adapt-univ generated more signals on average than adapt-univ-bic, adapt-bic and adapt-cisl. The latter generated approximately the same number of signals. For these three approaches, their number of signals was close to the number of true predictors.

**Fig. 3:**
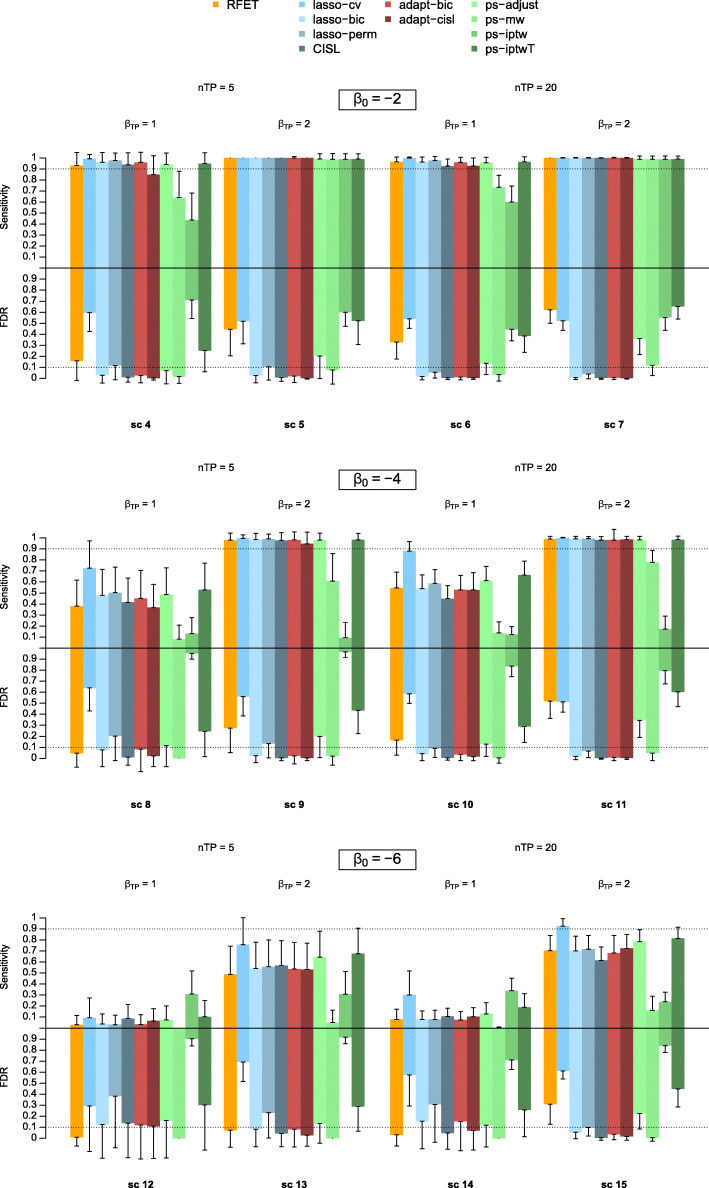
Sensitivity and False Discovery Rate of all signal detection approaches across scenarios 4 to 15. The upper and lower parts of the colour bars represent the average sensitivity and FDR of each approach over the 500 simulation replications respectively. The vertical solid lines extending the bars represent the standard deviation of the corresponding metrics

**Fig. 4:**
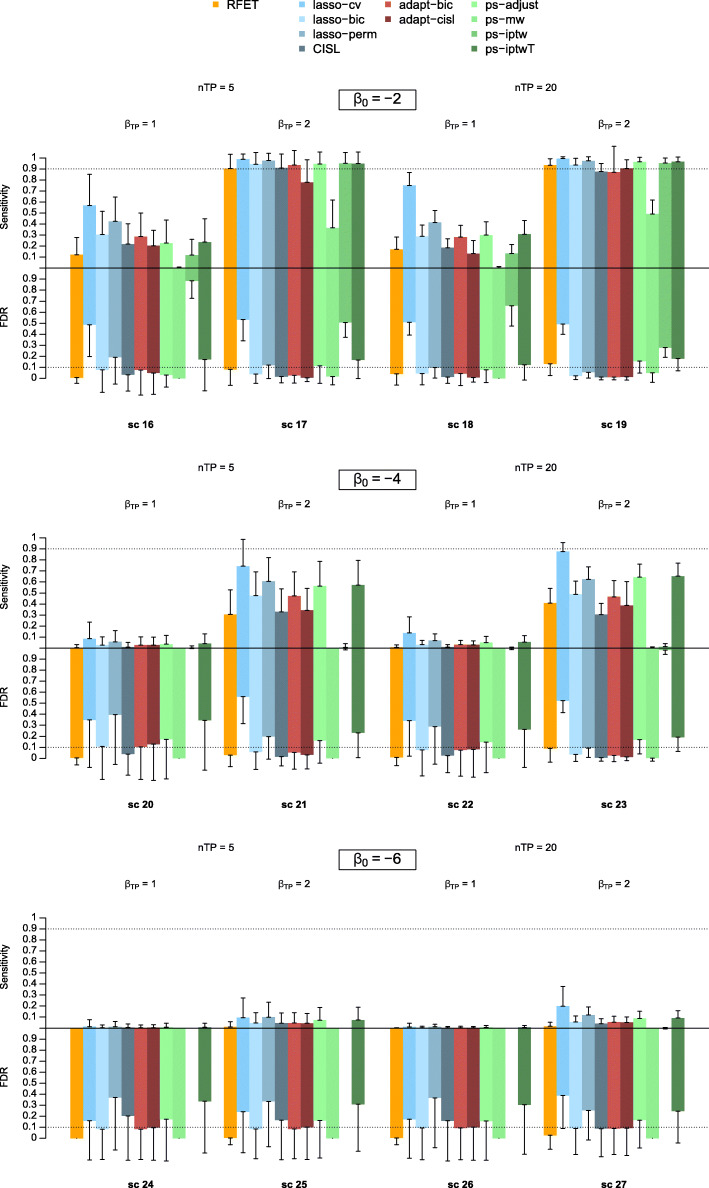
Sensitivity and False Discovery Rate of all signal detection approaches across scenarios 16 to 27. The upper and lower parts of the colour bars represent the average sensitivity and FDR of each approach over the 500 simulation replications respectively. The vertical solid lines extending the bars represent the standard deviation of the corresponding metrics

Since adapt-cisl and adapt-bic showed the best performances among the adaptive lasso-based methods, we retained only these two methods for the remaining comparisons with the other approaches. Figure 3 shows the average FDR and sensitivity of our two approaches, RFET, lasso-based and PS-based approaches when true predictors were reported more than 100 times (scenarios 4-15). Figure 4 shows the same results when true predictors were reported between 20 and 100 times (scenarios 16-27). Results for scenarios where there were no true predictors (1-3) are shown in Table 3. Lasso-cv, ps-iptwT had the best performances in terms of sensitivity but they both showed a high FDR across all the scenarios. To a lesser extent, RFET and ps-adjust showed the same behaviour in scenarios 7,11, 15 with an FDR up to 0.62 for RFET and up to 0.34 for adjust-ps. RFET also had this behaviour in scenarios 4 to 6. In all other scenarios, they both showed a rather low FDR. Overall, ps-adjust performed better than RFET both in terms of sensitivity and FDR in scenarios 4 to 15, but RFET reached a lower FDR in scenarios 16 to 27. On the other hand, ps-mw was very conservative: its FDR remained very low across the different scenarios, sometimes even much lower than the expected 5% fixed threshold. Its sensitivity dropped and became null as soon as the outcome became rarer and the true predictors reporting frequencies decreased (scenarios 12-15, 16, 18 and 20-27). The ps-iptw approach performed very poorly across all the scenarios with a very low sensitivity and an extremely high FDR. The lasso-based approaches other than lasso-cv showed good performances. Among them, lasso-perm performed worse with a high FDR when there were no true predictors (scenarios 1-3) with an FDR around 0.35, or when the outcome was rare, both for frequent and rare true predictors (scenarios 12-15 and 24-27) with an FDR up to 0.38. CISL and lasso-bic showed very good performances with both an acceptable sensitivity and a low FDR in most scenarios. When the outcome was rare, i.e. *β*_0_=−6, CISL showed an increase in its FDR. This increase was noticeable in scenarios 3, 12 and especially in scenarios 24 to 26, where CISL showed an FDR between 0.15 and 0.20. Overall, lasso-bic had a slightly higher sensitivity and FDR than CISL. Although lasso-bic had a fairly stable behaviour, it showed a surprising increase in its FDR in scenarios 14 and 20, with an FDR at 0.15 and 0.10, respectively.

**Table 3:** False Discovery Rate with standard deviation of all signal detection approaches across scenarios 1 to 3, e.g. for scenarios where *n*_*TP*_=0

Methods	False Discovery Rate		
	scenario 1: *β*_0_=−2	scenario 2: *β*_0_=−4	scenario 3: *β*_0_=−6
RFET	0.00 (0.00)	0.01 (0.09)	0.00 (0.04)
lasso-cv	0.31 (0.46)	0.28 (0.45)	0.12 (0.26)
lasso-bic	0.12 (0.33)	0.10 (0.30)	0.07 (0.26)
lasso-perm	0.37 (0.46)	0.41 (0.49)	0.35 (0.48)
CISL	0.06 (0.23)	0.04 (0.20)	0.18 (0.38)
adapt-bic	0.11 (0.32)	0.10 (0.30)	0.07 (0.26)
adapt-cisl	0.13 (0.33)	0.11 (0.32)	0.09 (0.29)
ps-adjust	0.01 (0.12)	0.15 (0.35)	0.14 (0.35)
ps-mw	0.00 (0.00)	0.00 (0.00)	0.00 (0.00)
ps-iptw	0.99 (0.10)	1.00 (0.00)	1.00 (0.00)
ps-iptwT	0.23 (0.42)	0.35 (0.48)	0.29 (0.45)

Compared to lasso-bic, our proposals showed an equivalent or lower sensitivity and a lower FDR in all scenarios. In particular, this difference in FDR was noticeable in scenarios 12 to 15 comparing adapt-cisl to lasso-bic. Like lasso-bic, adapt-bic and adapt-cisl showed an increase in terms of FDR for scenario 20 with an FDR of 0.12 for adapt-cisl and 0.10 for adapt-bic.

In supplementary materials, Table B and Table C show the average number of signals generated by RFET, the lasso-based and the PS-based approaches across all the scenario settings. The number of generated signals for all approaches considered here decreased with the scarcity of the outcome and true predictors. This was particularly the case for ps-mw which did not generate any signals for scenarios 20 to 27. All the approaches except ps-iptw had a low number of generated signals on average when *n*_*TP*_=0. For all other scenarios, the average number of signals generated was consistent with the observed performance in terms of true and false discoveries. Approaches such as lasso-cv, ps-iptwT, ps-iptw, RFET and ps-adjust generated too many signals compared to the number of true predictors, particularly when they were highly reported (scenarios 4-15). Lasso-bic, lasso-perm, CISL and to a lesser extent ps-mw, behaved like our proposals by generating a number of signals close to the number of true predictors across all the settings.

Lasso-based approaches showed larger standard deviation compared to other families of approaches when *n*_*TP*_=0. The stability of the results in terms of FDR decreased with the intercept value *β*_0_ as before, except for RFET and ps-mw. All approaches showed comparable stability results in terms of sensitivity, with an increase of the standard deviation when *β*_*TP*_=2.

## Real-world data analysis

### The french pharmacovigilance database

We applied the aforementioned signal detection approaches to the French pharmacovigilance data extracted from 1 January 2000 to 29 December 2017. We discarded spontaneous reports involving (i) drugs recorded as vaccines, phytotherapy, homeotherapy, dietary supplements, oligotherapy or enzyme inhibitors, (ii) reactions recorded as overdoses or medication errors. Drugs are listed according to their active substance which is coded with the 5th level of the Anatomical Therapeutic Chemical (ATC) hierarchy. AEs are coded according to the Preferred Term (PT) level of the Medical Dictionary for Regulatory Activities (MedDRA). This extraction of the French pharmacovigilance database included 452 914 reports with 6 617 different AEs and 2 378 different drugs.

### Comparison set-up

To assess the performances of these approaches, we used a reference signal set pertaining to the adverse event Drug-Induced Liver Injury (DILI) [28, 29]. The set was established by text-mining the FDA-approved drug labels with a list of keywords related to the DILI event. A level of DILI severity was assigned to each keyword: mild, moderate or severe DILI. According to where keywords appeared in the labelling section of the FDA-approved drug labels, drugs were classified in two DILI-related categories: “less-DILI-concern” and “most-DILI-concern”. If no keywords were found in the label, drugs were considered as “no-DILI-concern”. The majority of “most-DILI-concern” drugs were associated with severe DILI. This classification was refined later to assess the causal relationship between each drug and a DILI event using other data sources. Only drugs confirmed as a cause of DILI were retained. We translated the list of keywords used to define a DILI event into Preferred Terms (PT) codes from the MedDRA classification. If a spontaneous report involved at least one of the PT codes, it was considered as a reported DILI event. This resulted in considering 25 187 DILI reports in the French pharmacovigilance database. We considered the “no-DILI-concern” drugs as true negatives, and the “most-DILI-concern” drugs as true positives.

Over the study period, the database consisted of 1 692 different drugs reported more than 10 times. Among these drugs, 1 136 had more than three reports in common with a DILI. As in the simulation work, RFET and all the PS-based signal detection approaches were implemented on these 1 136 drugs and the remaining 556 had their p-value set to one. In the end, the DILI reference signal set contained 203 true negative controls and 133 true positive controls among the 1 692 drugs. Of the 1 136 drugs retained after filtering, the reference signal set contained 123 true negative controls and 119 true positive ones.

### Results

Table 4 summarizes the results of all the methods in terms of generated signals, False Discovery Proportion (FDP), specificity and sensitivity derived from the DILI reference signal set. Despite the wide variability in terms of number of generated signals, we observe that 10 methods out of 14 achieved a rather comparable balance between false positives and sensitivity as regards the reference set. As in the simulations, adapt-cisl and adap-bic showed good performance in terms of false discoveries, at the cost of lower sensitivity. Some methods such as lasso-cv and adapt-univ showed better performance than in the simulations. Among all the compared methods, adapt-cisl showed the best performances with only two false positives out of 60 signals with known status.

**Table 4:** Performance of each method in terms of number of signals, False Discovery Proportion (FDP), specificity and sensitivity. Operating characteristics are calculated based on drugs with known status

Method	Number of	Number of signals	Number of false	FDP	Specificity	Sensitivity
	generated signals	with known status	positive signals	(%)	(%)	(%)
		(positive or negative)				
RFET	249	82	13	15.9	93.6	51.9
lasso-cv	220	79	5	6.3	97.5	55.6
lasso-bic	170	65	5	7.7	97.5	45.1
lasso-perm	158	60	4	6.7	98.0	42.1
CISL	109	48	2	4.2	99.0	34.6
adapt-cv	188	70	5	7.1	97.5	48.9
adapt-univ	179	65	4	6.2	98.0	45.9
adapt-univ-bic	163	61	4	6.6	98.0	42.9
adapt-bic	151	60	4	6.7	98.0	42.1
adapt-cisl	153	60	2	3.3	99.0	43.6
ps-adjust	209	71	6	8.5	97.0	48.9
ps-mw	86	40	0	0.0	100.0	30.1
ps-iptw	49	15	3	20.0	98.5	9.0
ps-iptwT	260	84	12	14.3	94.1	54.1

**Fig. 5:**
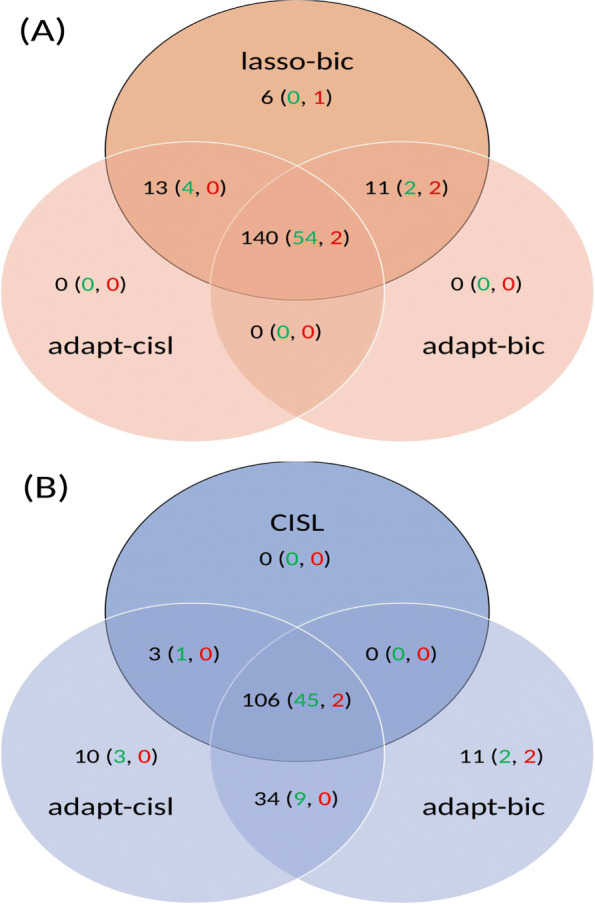
Distribution of signals generated by adapt-cisl, adapt-bic and **(A)** lasso-bic; **(B)** CISL. Among signals generated, true positives are in green and false positives in red

Figure 5-A shows the overlap between signals generated by adapt-cisl, adapt-bic and lasso-bic and Fig. 5-B shows this overlap for adapt-cisl, adapt-bic and CISL. Among the signals generated, true positives and false positives according to the reference set are also represented. Figure 5-A shows that all the signals generated by our two proposals were also generated by lasso-bic: 140 signals were common to the three methods, 13 were generated by adapt-cisl and lasso-bic, and 11 were generated by adapt-bic and lasso-bic. Six signals were generated by lasso-bic only, of which none were known to be positive and one was a known negative. Among the signals generated only by adapt-cisl or adapt-bic and common to lasso-bic, adapt-cisl generated four true signals and no false positive, whereas adapt-bic was a little less efficient with two true positives and two false positives. There were 54 true positives and two false positives among the 140 signals generated by the three methods. Figure 5-B shows that all signals generated by CISL were also generated by adapt-cisl with 106 signals common for the three methods (CISL, adapt-cisl, adapt-bic), three in common between CISL and adapt-cisl, and 10 additional signals generated by adapt-cisl only with three true positives and no false positives. Adapt-bic did not share any signals with CISL only and it generated 11 signals on its own with two true positives and two false positives, i.e. the same generated by lasso-bic. Overall, adapt-cisl performed well since its only two false positives among associations with known status were shared with the three other methods and no additional false positives occurred by itself. This was not the case lasso-bic and adapt-bic.

## Discussion

The development of novel signal detection methods is crucial for improving the responsiveness and the efficiency of post-marketing surveillance systems. In this work we propose new approaches for signal detection based on an appropriate methodology for variable selection: the adaptive lasso. In addition to defining new adaptive penalty weights derived from lasso-based approaches, we used the BIC to perform variable selection. To assess the performances of our strategies, we performed an extensive simulation study conducted for multiple scenario configurations and an application to real data, where we compared our approaches to other implementations of the adaptive lasso in high dimension found in the literature, as well as to other detection approaches recently proposed based either on lasso regression or on PSs. Methods for signal detection in pharmacovigilance must both be able to avoid time-wasting false positive signals in the context of further assessment resource constraints, and they must also not miss true positive signals for obvious public health issues. Thus, we chose in this work to evaluate our methods using the two criteria of sensitivity and FDR. We developed an R package available on CRAN that implement all the methods compared in the present work.

By comparing all the adaptive lasso-based approaches including our two proposals, adapt-bic and adapt-cisl, we first demonstrate that our defined AWs and the use of the BIC for variable selection are relevant for signal detection. Cross-validation for the adaptive lasso is a computationally intensive procedure since it requires deriving adaptive weights for each training set, and shows an unstable behaviour in terms of detection. The broader comparison that includes state-of-the-art signal detection approaches shows that our proposals are particularly competitive.

Compared to lasso regression where BIC is used to perform variable selection, an approach we called lasso-bic here, our proposals tend to show a lower FDR at the cost of a slightly lower sensitivity. For adapt-bic, this result is not surprising since by construction, the covariates selected by this approach are a sub-sample of the covariates selected by the lasso-bic approach.

Our work also confirms that CISL is a relevant signal detection approach. The choice of the quantile, which we set at 10%, seems appropriate for a large number of settings, except when the outcome and true predictors are rare. As expected, cross-validation is not appropriate for signal detection. All the approaches based on this criterion: lasso-cv, adapt-cv and adapt-univ show a high sensitivity at the cost of a very high FDR in the vast majority of scenarios. The use of the permutation method with lasso regression (lasso-perm) does not show fully satisfactory results with an FDR generally higher than the one of our proposals and a moderate gain in sensitivity.

Among the PS-based approaches for signal detection, results are concordant with our previous work [17]. Our simulation study shows that weighting on the propensity score with matching weights perform very well when the outcome was frequent but become very conservative with a substantial drop in sensitivity as the outcome became rarer. This is a disadvantage since it is common in pharmacovigilance datasets to have very few reported outcomes. Adjustment on the PS lead to a high sensitivity and a quite high FDR among several simulation settings. The ps-iptw approach showed very poor performances in all settings. As discussed in our previous work, these results can be explained by a potential numerical instability of weights, as already reported in the literature [38]. Performing truncation of those weights improves these results, but it is still an unsatisfactory signal detection approach since it leads to a significant number of false discoveries.

Overall, our approaches show very satisfactory performances in terms of false discoveries and a good sensitivity. This behavior remains stable over all simulation scenarios, with a slight increase in FDR when there are no true predictors. Among our two proposals, adapt-cisl performs slightly better. Lasso-bic and CISL are also relevant detection approaches, with the few nuances in terms of performance detailed above. RFET and to a lesser extent ps-adjust provide sensitivity that is sometimes superior to our proposals but with a fluctuating FDR which can be high, especially when the number of predictors increases and are strongly associated with the response. Finally, making the concession of a large number of false discoveries (and thus a large number of signals to review), lasso-cv is the approach that provides the best sensitivity in all situations.

It is more difficult to assess the differences in performances of the approaches from the results of the application to real data. As the DILI adverse event is highly reported and since the majority of drugs registered in the French pharmacovigilance database are much less reported, the most comparable simulation scenarios to this situation are scenarios 16 to 19. The behaviour of the approaches differs slightly from that observed in simulation, but among all the approaches tested, our adapt-cisl approach showed the best compromise between a very low FDR, an acceptable sensitivity and a reasonable number of generated signals.

Using the BIC as a criterion to select the penalisation parameter in lasso regression for variable selection has been widely studied [39–42]. In particular, Chen and Chen [43] defined the extended Bayesian Information Criterion (eBIC), which is suitable for model selection in large model spaces. With this criterion, a term is added to the original BIC to correct for the prior probability of the different possible models in order to promote small dimension models. The BIC is a particular case of the eBIC. Chen and Chen showed that this criterion is particularly relevant in the large-P-small-N configuration. Considering that here we are in the *P*<<*N* situation, implementing the original BIC to perform variable selection seemed reasonable. In the case of the adaptive lasso, Hui et al. [44] developed the Extended Regularized Information Criterion (ERIC) to perform variable selection. They argued that the BIC cannot account for the prior information carried by the AWs. In their work, they considered the BIC defined with the penalised log likelihood. By using the BIC which is based on the unpenalized likelihood, we avoid some of the issues raised by Hui et al. However, it would now be interesting to compare the variable selection for the adaptive lasso operating with the original BIC versus ERIC in our context.

To preserve the specificities of pharmacovigilance datasets, i.e. a large size and sparsity, we based our simulation study on real data. This strategy has already been used in the literature to simulate large health care data [34]. We varied the number and the frequency of true predictors and their strength of association with the outcome, the rarity of which we also varied. With this strategy, it was not possible to vary the correlation structure between the true predictors and the other variables. However, we were able to evaluate the performance of our detection methods with a realistic correlation structure between the variables. As regard to the performance of our proposals, we are rather confident that our methods manage satisfactorily the correlation between variables. An extension of this work would be to develop a more complex strategy for simulating data that would provide correlation structures that we could control while remaining realistic. Nevertheless, defining such a set of realistic correlation structures is a challenging task.

A major issue in the development of signal detection methods is the lack of reliable and sufficiently large sets of reference signals to evaluate performance in real-life conditions. Here we considered a set of reference signals pertaining to a common adverse event: DILI. Although this set is very broad, it still has its limitations. Indeed, the performances of the approaches are more difficult to interpret since they can only be evaluated on signals whose status has been assessed. It would be interesting to extend this application to other adverse events.

## Conclusion

The simulation and the application results suggest that the adaptive lasso is an appealing methodology for pharmacovigilance when the adaptive penalty weights are cleverly chosen, and when an appropriate variable selection criterion is used. Although the BIC does not make it possible to control the FDR, we are confident that it is relevant in view of the results of our simulations. Finally, our approaches do not require much more computation time than lasso-based approaches, and take far less time than that needed for PS-based approaches. An interesting development could consist in integrating external relevant information through adaptive penalty weighting in the pharmacovigilance context. Under the weighted lasso designation, this technique has proved to be very attractive in an area such as genomics for improving penalised regression performances in terms of prediction and variable selection [45, 46].

## Supplementary Information

**Additional file 1** Supplementary materials : average number of signals generated across simulation scenarios Average number of signals generated by adaptive lasso-based approaches (Table A), RFET and lasso-based approaches (Table B) and PS-based approaches (Table C) across all simulated scenarios

## Data Availability

The datasets generated and analysed during the current study are not publicly available due to ethical restrictions but are available from the corresponding author on reasonable request.
