# Supplementary material for: New adaptive lasso approaches for variable selection in automated pharmacovigilance signal detection
Source: BMC Med Res Methodol. 2021 Dec 1;21:271. doi: 10.1186/s12874-021-01450-3 (PMC8638444; doi:10.1186/s12874-021-01450-3)
Supplement: Supplementary file 1 — Additional file 1 Supplementary materials : average number of signals generated across simulation scenarios Average number of signals generated by adaptive lasso-based approaches (Table A), RFET and lasso-based approaches (Table B) and PS-based approaches (Table C) across all simulated scenarios [file 12874_2021_1450_MOESM1_ESM.pdf]

Supplementary Material: New adaptive lasso approaches for variable selection in automated pharmacovigilance signal detection

Émeline Courtois*[,1], Pascale Tubert-Bitter †[,1], and Ismaïl Ahmed ‡[,1]

[1]Inserm, Université Paris Saclay, Inserm U1018 - Center for Epidemiology and Population Health (CESP), Team High-Dimensional Biostatistics for Drug Safety and Genomics.

*Corresponding author: emeline.courtois@inserm.fr
†These authors share last authoship
‡

Scenarios	β_0	n_{TP}	β_{TP}	Methods				
				adapt-cv	adapt-univ	adapt-univ-bic	adapt-bic	adapt-cisl
No true predictors								
1	-2	0	0	0.12	0.21	0.07	0.12	0.13
2	-4	0	0	0.33	0.05	0.06	0.11	0.12
3	-6	0	0	0.32	0.01	0.05	0.08	0.09
True predictors reported more than 100 times								
4	-2	5	1	6.57	12.06	4.96	4.95	4.25
5	-2	5	2	5.34	8.06	5.09	5.12	5.00
6	-2	20	1	23.03	37.36	19.65	19.42	18.61
7	-2	20	2	22.4	28.53	20.14	20.11	20.01
8	-4	5	1	6.86	15.68	2.53	2.47	1.89
9	-4	5	2	7.56	11.85	5.12	5.04	4.75
10	-4	20	1	26.67	49.07	11.65	11.00	10.77
11	-4	20	2	25.88	36.91	20.43	19.75	19.75
12	-6	5	1	1.31	0.03	0.18	0.34	0.47
13	-6	5	2	4.08	1.04	2.99	2.98	2.76
14	-6	20	1	4.58	0.18	1.23	1.92	2.28
15	-6	20	2	5.92	3.34	14.85	14.19	14.74
True predictors reported between 20 and 100 times								
16	-2	5	1	1.94	6.04	1.45	1.57	1.09
17	-2	5	2	5.43	8.21	4.78	4.82	3.92
18	-2	20	1	13.03	23.76	5.69	5.87	2.68
19	-2	20	2	22.69	27.77	18.78	17.62	18.35
20	-4	5	1	0.63	0.12	0.21	0.27	0.27
21	-4	5	2	2.50	0.86	2.47	2.53	1.79
22	-4	20	1	1.32	0.20	0.57	0.74	0.71
23	-4	20	2	7.46	2.88	9.97	9.56	7.89
24	-6	5	1	0.57	0.01	0.08	0.11	0.12
25	-6	5	2	1.07	0.05	0.29	0.33	0.34
26	-6	20	1	0.56	0.02	0.11	0.17	0.17
27	-6	20	2	3.84	0.10	1.13	1.25	1.19

Table A: Average number of signals generated by adaptive lasso-based approaches across all simulated scenarios.

Scenarios	β_0	n_{TP}	β_{TP}	Methods				
				RFET	lasso-cv	lasso-bic	lasso-perm	CISL
No true predictors								
1	-2	0	0	0.00	1.41	0.13	0.55	0.06
2	-4	0	0	0.01	1.37	0.12	0.63	0.04
3	-6	0	0	0.00	0.70	0.08	0.55	0.20
True predictors reported more than 100 times								
4	-2	5	1	5.91	14.70	4.97	5.65	4.74
5	-2	5	2	11.81	12.53	5.16	5.70	5.05
6	-2	20	1	30.51	45.02	19.58	20.74	18.61
7	-2	20	2	57.84	43.17	20.15	20.85	20.07
8	-4	5	1	2.09	13.50	2.64	3.31	2.10
9	-4	5	2	7.87	13.57	5.07	5.86	4.90
10	-4	20	1	13.77	44.39	11.33	13.02	9.00
11	-4	20	2	45.58	42.61	20.17	21.34	19.64
12	-6	5	1	0.17	3.49	0.38	0.86	0.65
13	-6	5	2	2.83	15.82	3.01	3.82	2.99
14	-6	20	1	1.74	21.17	2.05	2.53	2.26
15	-6	20	2	22.73	49.24	14.89	15.99	12.34
True predictors reported between 20 and 100 times								
16	-2	5	1	0.63	8.51	1.69	2.81	1.15
17	-2	5	2	5.13	12.64	4.93	5.66	4.63
18	-2	20	1	3.68	32.46	6.03	9.25	3.75
19	-2	20	2	21.89	40.44	19.16	20.68	17.71
20	-4	5	1	0.02	2.82	0.28	1.00	0.08
21	-4	5	2	1.63	11.70	2.55	3.92	1.68
22	-4	20	1	0.17	7.29	0.75	2.18	0.22
23	-4	20	2	9.28	38.93	10.14	13.84	6.13
24	-6	5	1	0.00	1.19	0.11	0.70	0.25
25	-6	5	2	0.06	2.34	0.33	1.21	0.42
26	-6	20	1	0.01	1.13	0.17	0.88	0.23
27	-6	20	2	0.45	10.29	1.28	3.32	0.94

Table B: Average number of signals generated by RFET and lasso-based approaches across all simulated scenarios.

Scenarios	β_0	n_{TP}	β_{TP}	Methods			
				ps-adjust	ps-mw	ps-iptw	ps-iptwT
No true predictors							
1	-2	0	0	0.02	0.00	4.73	0.26
2	-4	0	0	0.18	0.00	13.93	0.46
3	-6	0	0	0.16	0.00	14.07	0.38
True predictors reported more than 100 times							
4	-2	5	1	5.17	3.27	7.96	6.95
5	-2	5	2	6.84	5.48	13.67	13.55
6	-2	20	1	22.47	15.24	22.08	33.37
7	-2	20	2	32.59	22.64	47.43	62.30
8	-4	5	1	2.86	0.41	14.02	3.79
9	-4	5	2	6.65	3.14	13.84	10.54
10	-4	20	1	14.39	2.77	15.03	19.73
11	-4	20	2	31.90	16.46	16.29	55.09
12	-6	5	1	0.65	0.00	16.08	1.11
13	-6	5	2	3.94	0.25	18.88	5.41
14	-6	20	1	3.13	0.03	23.65	5.35
15	-6	20	2	21.18	3.24	30.62	32.86
True predictors reported between 20 and 100 times							
16	-2	5	1	1.21	0.00	5.46	1.58
17	-2	5	2	5.60	1.89	10.29	6.01
18	-2	20	1	6.67	0.04	7.87	7.24
19	-2	20	2	23.26	10.46	26.83	24.00
20	-4	5	1	0.43	0.00	13.88	0.73
21	-4	5	2	3.57	0.00	13.89	3.96
22	-4	20	1	1.44	0.00	13.84	1.84
23	-4	20	2	15.95	0.03	13.93	16.67
24	-6	5	1	0.24	0.00	13.64	0.50
25	-6	5	2	0.61	0.00	13.83	0.89
26	-6	20	1	0.32	0.00	13.89	0.56
27	-6	20	2	2.37	0.00	14.76	2.74

Table C: Average number of signals generated by PS-based approaches across all simulated scenarios.
